# Peer Review of “Left Ventricular Outflow Tract Obstruction in Patients Treated With Milrinone for Cerebral Vasospasm: Case Report and Literature Review”

**DOI:** 10.2196/37032

**Published:** 2022-04-11

**Authors:** Sunil Munakomi

**Affiliations:** 1 Department of Neurosurgery College of Medical Sciences Bharatpur Nepal

**Keywords:** ventricular outflow obstruction, subarachnoid hemorrhage, vasospasm, intracranial, milrinone, hemorrhage, neurosurgery, neurology, surgery, pharmaceutical

*This is a peer-review report submitted for the paper “Left Ventricular Outflow Tract Obstruction in Patients Treated With Milrinone for Cerebral Vasospasm: Case Report and Literature Review.”*

## Review Round 1

This paper [1] deals with a rare event on the occurrence of left ventricular outflow obstruction in a patient treated with milrinone for vasospasm following an aneurysmal bleed.

### Major Comments

1. The rationale for radioembolization of aneurysms needs to be elaborated.
2. The probable differential diagnosis of stunned myocardium syndrome in the acute phase needs to be mentioned.
